# Exploration of Sports Participation and Curriculum Resource Utilization in Primary Schools Before and After the “Double Reduction”

**DOI:** 10.3389/fpsyg.2022.898675

**Published:** 2022-07-15

**Authors:** Shuhong Liu, Guihong Wang

**Affiliations:** ^1^School of Physical Education, Liaoning Normal University, Dalian, China; ^2^Graduate Students' Affairs Department, Shenyang Sport University, Shenyang, China; ^3^School of Social Sports, Shenyang Sport University, Shenyang, China

**Keywords:** Double Reduction policy, sports participation, students, empirical research, questionnaire survey

## Abstract

In order to eliminate capital chaos in the Education And Training (EAT) industry and ease parents' and students' excessive attention to subject achievements over physical quality, China government has launched the “Double Reduction” (“DR”) policy which promotes students' Sports Participation (SP) in the Compulsory Education (CE) stage concerning students' physical and mental health. Firstly, based on the actual situation of students' SP before and after releasing the “DR” policy, this paper understands the exact needs of parents and children. Secondly, following empirical research and mathematical statistics, it analyzes the structure and characteristics of students' SP before and after the release of the “DR” policy. Mainly, the experiment focuses on the frequency, project types, and off-campus class expenditure. It also considers students' SP motivation in on-campus and off-campus sports classes before and after the “DR” policy proposal. Additionally the general curriculum resource utilization of PE teachers are surveyed in order to find out the current status of on-campus PE classes. Eventually, the strategies are put forward to optimize students' SP under the “DR” from the perspectives of family, school, and society. The results show that after the release of “DR”, parents and schools gradually pay attention to student's physical health and better understand students' physical exercise in school. The consumption expenditure on sports off-campus classes has increased significantly. Meanwhile, family income and the father's occupation significantly impact the children's SP frequency in off-campus sports classes. Overall, “DR” is a protracted war. The existing difficulties need to be solved by families, schools, and the government. The research provides a practical basis for extending and managing on-campus sports classes and training. It helps timely uncover the problems in policy implementation. It guides the formulation of PE policy in the next stage of CE.

## Introduction

Since the founding of the People's Republic of China (PRC), the field of basic education in China has undergone five burden reduction orders to solve the physical and mental health problems of the primary and secondary school students in the stage of Compulsory Education (CE), in 1955, 2000, 2013, 2018, and 2021, respectively. Specifically, in 1955, educational reform reduced the burden thrice, followed by list reduction. Then, 2021 has seen a most thorough, rigorous, and explosive student burden reduction through root causes (Petrie et al., 2018; Zhang et al., 2020; Neville and Makopoulou, 2021). On May 21, 2021, at the 19th meeting of the Central Commission for Comprehensively Deepening Reform, the Opinions On Double Reduction were formally adopted for deliberation. On July 24, the general office of the Communist Party of China (CPC) Central Committee and the general office of the State Council officially issued the opinions on further reducing the burden of homework and after-school training of students in the stage of CE. In this way, with high specification, short time, and fine content, the Double Reduction (DR) work has hammered into the field of education and pried the change in the education ecosystem. The document jointly issued by the CPC Central Committee and the State Council has also effectively proved the importance of “DR”.

In order to strengthen the physical health education of primary school students and improve their comprehensive quality, on September 8, the Ministry of Education systematically held a series of press conferences on the golden autumn of 2021 education. The conference summarizes the existing problems in school Physical Education (PE) and discloses relevant data. About 22% of school PE and health courses in China are insufficient, and the cultural courses frequently occupy PE courses. In 2019, the excellent and good rate of physical health of students aged 6–12 was only 18.8% (Levinson et al., 2020). Therefore, the physical health level of teenagers in China needs to be improved (Goncharova, 2018; Lang, 2021; Papastergiou et al., 2021). The “DR” policy also highlights the importance of sports. The Ministry of Education promotes the “double increase” through the “DR” policy proposal. The “one increase” is to reduce the burden of schoolwork in the school and free up more time and opportunities for students to participate in outdoor activities, physical exercise, and artistic activities. The “other increase” lists sports and esthetic education training as non-discipline training. In essence, it increases students' time and opportunities to receive extra-curricular training in sports and aesthetic education (Moeijes et al., 2019; Bobrowski, 2021; Ilhan et al., 2021; Nathan et al., 2021). The “DR” policy has helped to develop physical education classes and students' Sports Participation (SP), but the real effectiveness of the policy lies in the effective implementation of PE, so this aspect of PE is particularly important. Among many contemporary theories of teaching and learning, the generative teaching theory conforms to the requirements of the development of the times for educational work, pays attention to students' differences, meets different needs and takes students as the main body, and emphasizes not only the development of students' physical health but also the strengthening of students' mental health development (Wang et al., 2020; Harvey and Smal, 2021; He et al., 2021). The Development And Utilization (DAU) of Generative Curriculum Resources (GCR) is the key link for teachers to carry out generative teaching. Teachers' active development of GCR can directly affect the overall development of students (Bosquet et al., 2020; Cárcamo et al., 2021). However, few studies utilize GCRorSports Participation (SP) in primary school PE.

Shenyang is one of the pilot cities for implementing the “DR” policy. What is the current status of on-campus PE activities? Do parents realize the importance of children's physique and sign up for off-campus sports training courses? What kinds of sports activities do parents tend to sign up for their children? These questions are all expounded on in the present work. First, this paper analyzes the GCR DAU of humanistic sports in primary schools and the actual situation of students' SP before and after introducing the “DR” policy. Then, it understands the actual needs of different parents and children and provides a certain practical basis for extending the role of on-campus sports and the management of extra-curricular sports training. The outcome points out the problems existing in implementing the policy in time and promotes and guides the formulation of PE policies in the next stage of CE.

## Methods

### Research Object and Method

The formal questionnaire (QS) is distributed to the parents of students in five primary schools in the pilot city of Shenyang through convenience sampling from November 1 to November 18, 2021. The respondents are parents of students in grades 2–6 of primary school. The QS data are collected, screened, sorted, and analyzed by Excel and Statistical Science for Social Science (SPSS). A total of 1,240 QSs are distributed, 1,098 QSs are collected, with a recovery rate of 88%, 956 valid QSs, and an effective rate of 87%. Additionally, this work also interviews 38 PE teachers to understand the utilization of GCR in primary school before and after the “DR” policy.

### QS Design and Preparation

The QS is designed. The aim is to understand the situation and changes in students' SP since implementing the “DR” policy and selecting sports items by students from families of different social classes under the current upsurge of off-campus sports training. QS includes a survey introduction, basic information about students and their families (namely, students' gender, number of family children, parents' age, income, occupation, and education level), frequency of students' SP in on-campus sports activities, types of sports activities, frequency of SP in off-campus physical education, cost of off-campus PE and motivation for admission. The respondents are parents of students in grades 2–6 of primary school (aged 6–12 years).

### Statistics of Sample Frequency Distribution

The sample FD statistics are given in Table 1.

**Table 1:** Statistics of sample FD.

**Variable**	**Category**	**Percentage/%**
Gender	Male	55.4%
	Female	44.5%
Only-child situation	Only-child	67.9%
	Multi-children	32.1%
Maternal occupation	Workers or the unemployed	15.6%
	Service personnel or individual industrial and commercial households	34.4%
	Civil servants of Party and government organs, personnel of enterprises and institutions, or technicians	27.6%
	Teachers, engineers, doctors, and lawyers	19.7%
	Other	2.7%
Father's occupation	Workers or the unemployed	13.6%
	Service personnel or individual industrial and commercial households	34.8%
	Civil servants of Party and government organs, personnel of enterprises and institutions, or technicians	32.2%
	Teachers, engineers, doctors, and lawyers	16.6%
	Other	2.7%
Mother's education	Lower than high school	28.4%
	Diploma or undergraduate	62.2%
	Post-graduate	9.4%
Father's occupation	Lower than high school	29.3%
	Diploma or undergraduate	59.2%
	Post-graduate	11.5%

As manifested in Table 1, the QS sample is rich. The structure is reasonable, proving the good representativeness of the QS through the gender, one-child situation, and parents' educational background and occupational structures of the respondents.

## Results

### Analysis of the DAU of GCR of Primary School PE

#### The Main Motivation of PE Teachers to Develop and Utilize GCR

This section investigates the respondents through direct questions and interviews. It analyzes the main motivation of PE teachers to develop and use GCR. Table 2 lists the results. Q1: “what is your main motivation to develop and use GCR in teaching?” Q2: “what do you think are the main factors to evaluate teachers' teaching ability?”

**Table 2:** Interview data on the main motivation of PE teachers to develop and utilize GCR.

**Questions**	**Teachers' answers**	**Proportion**
Q1	To develop students' emotions, attitudes, and values	5.3%
	To develop students' thinking	7.9%
	To help students improve their movement skills	21.1%
	To achieve the preset teaching objectives	65.8%
Q2	Development of students' thinking ability	7.9%
	Students' emotions, attitudes, and value development	2.6%
	Mastery of students' skills	28.9%
	Completion of teaching plan	50.0%
	Classroom discipline	10.5%

As listed in Table 2, 65.8% of teachers develop and utilize GCR to achieve the present teaching objectives. Regarding the main factors to evaluate teachers' teaching ability, 50% of teachers choose “the completion of the teaching plan”, and 28.9% of teachers choose the “mastery of students' skills”. Overall, in practice, most teachers always regard “the completion of teaching plans” as the purpose and destination of teaching activities, ignoring the needs of students' development of emotion, attitude, and values.

#### DAU of Different Types of GCR

**Figure 1:**
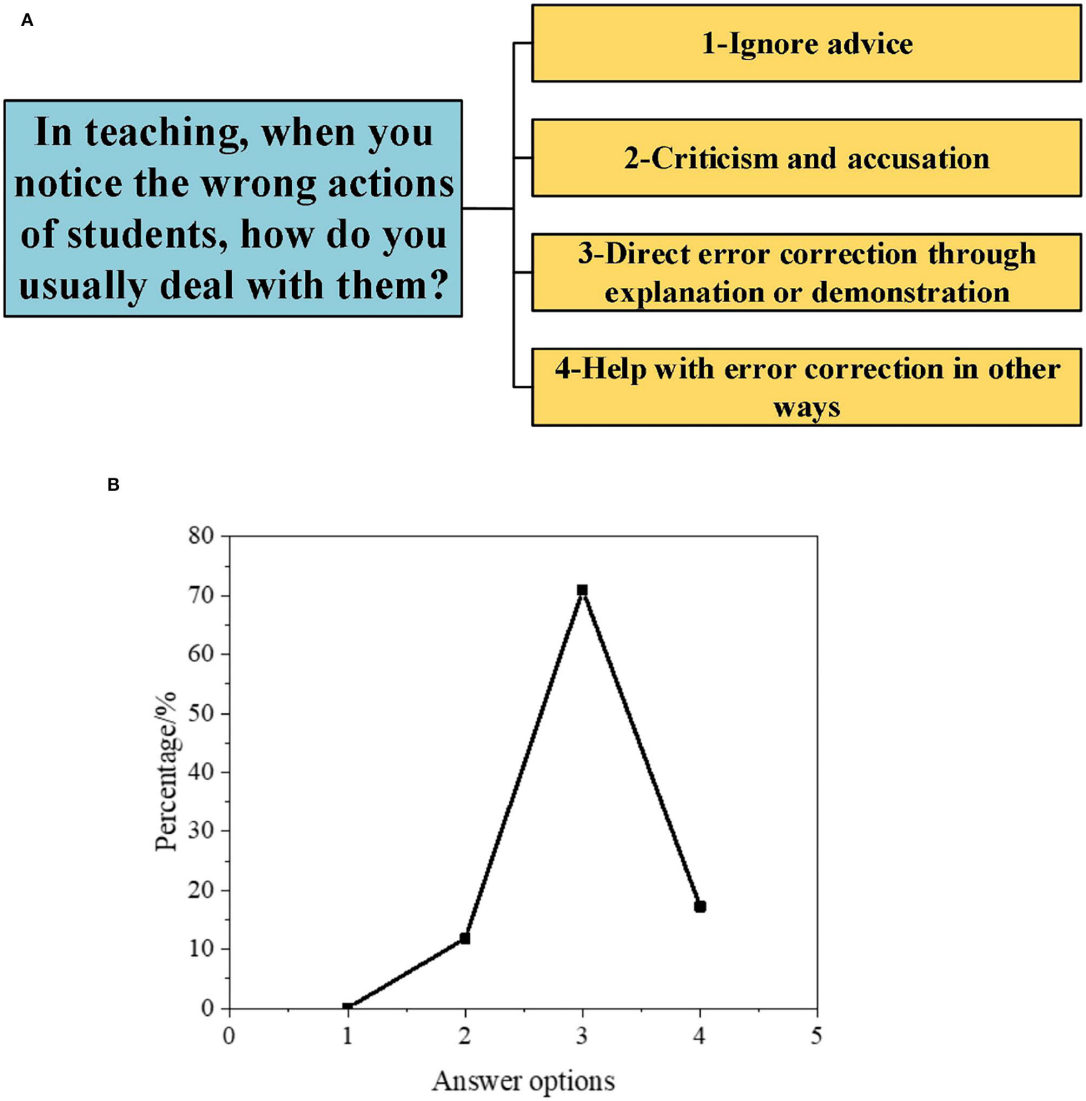
Interview data on the DAU of students' error-oriented GCR [**(A)**: questions and options; **(B)**: proportion of options].

**Figure 2:**
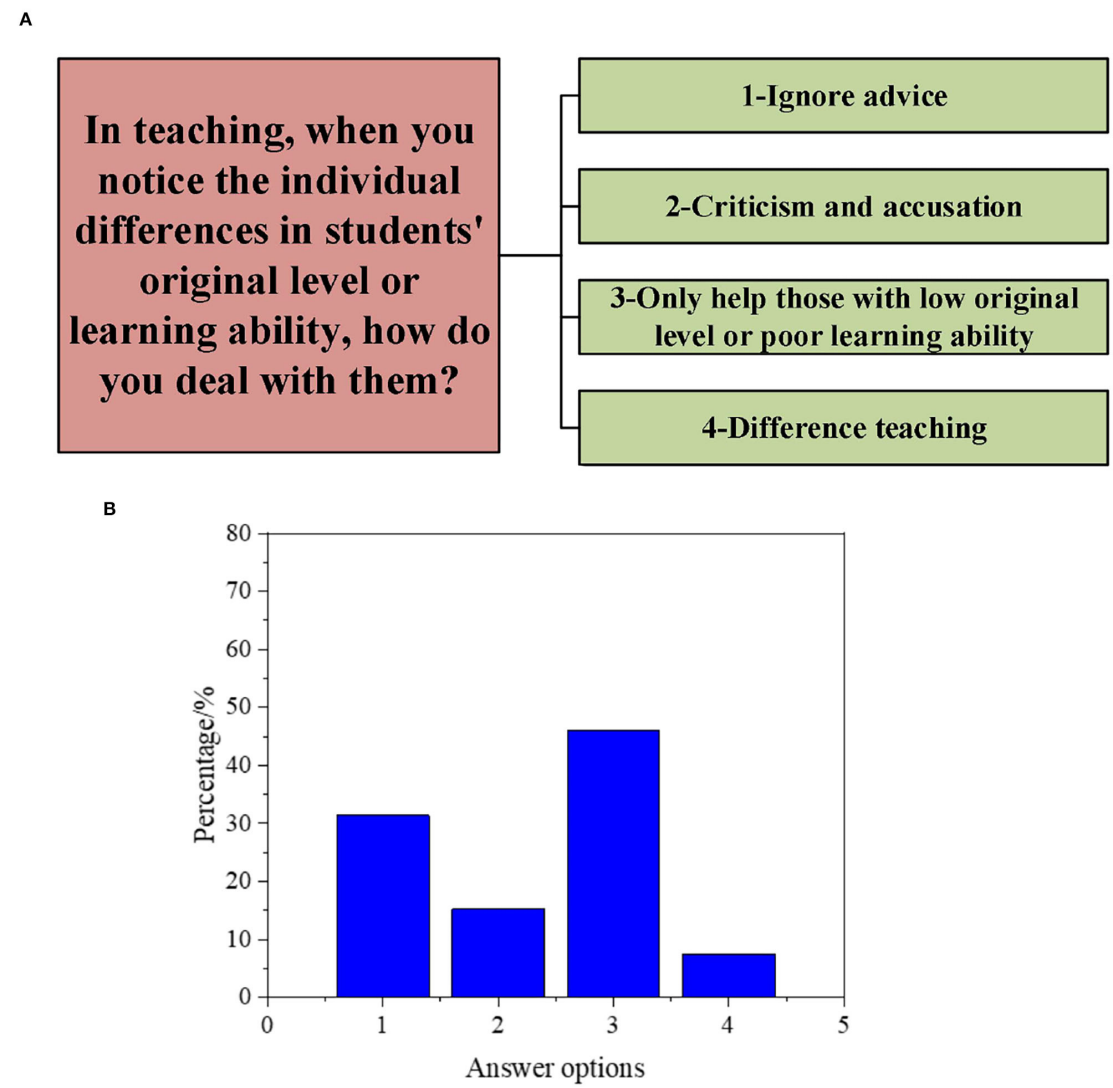
Interview data on students' individual difference-oriented GCR [**(A)**: questions and options; **(B)**: proportion of options].

**Figure 3:**
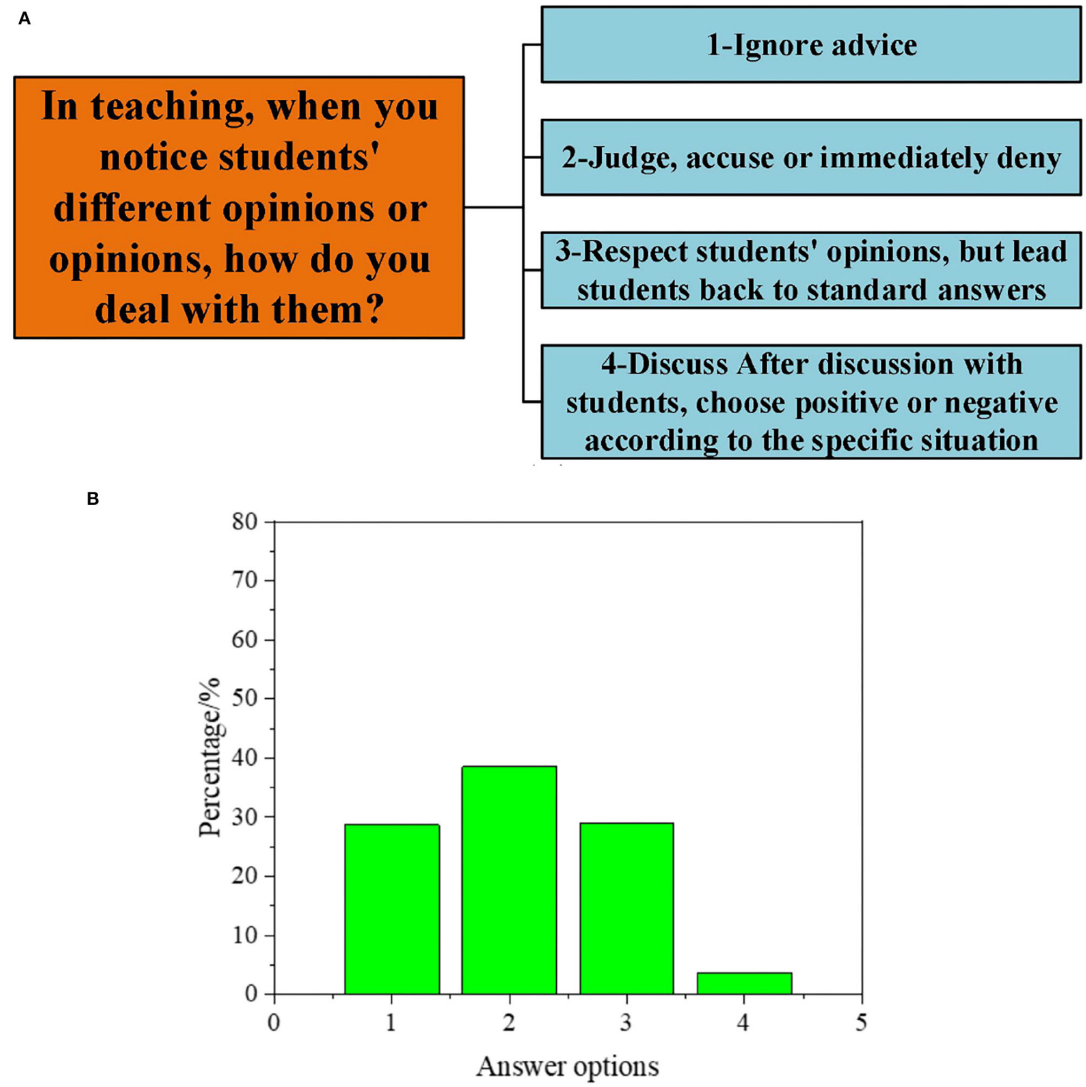
Interview data on the DAU of students' suggestion-oriented GCR [**(A)**: questions and options; **(B)**: proportion of options].

**Figure 4:**
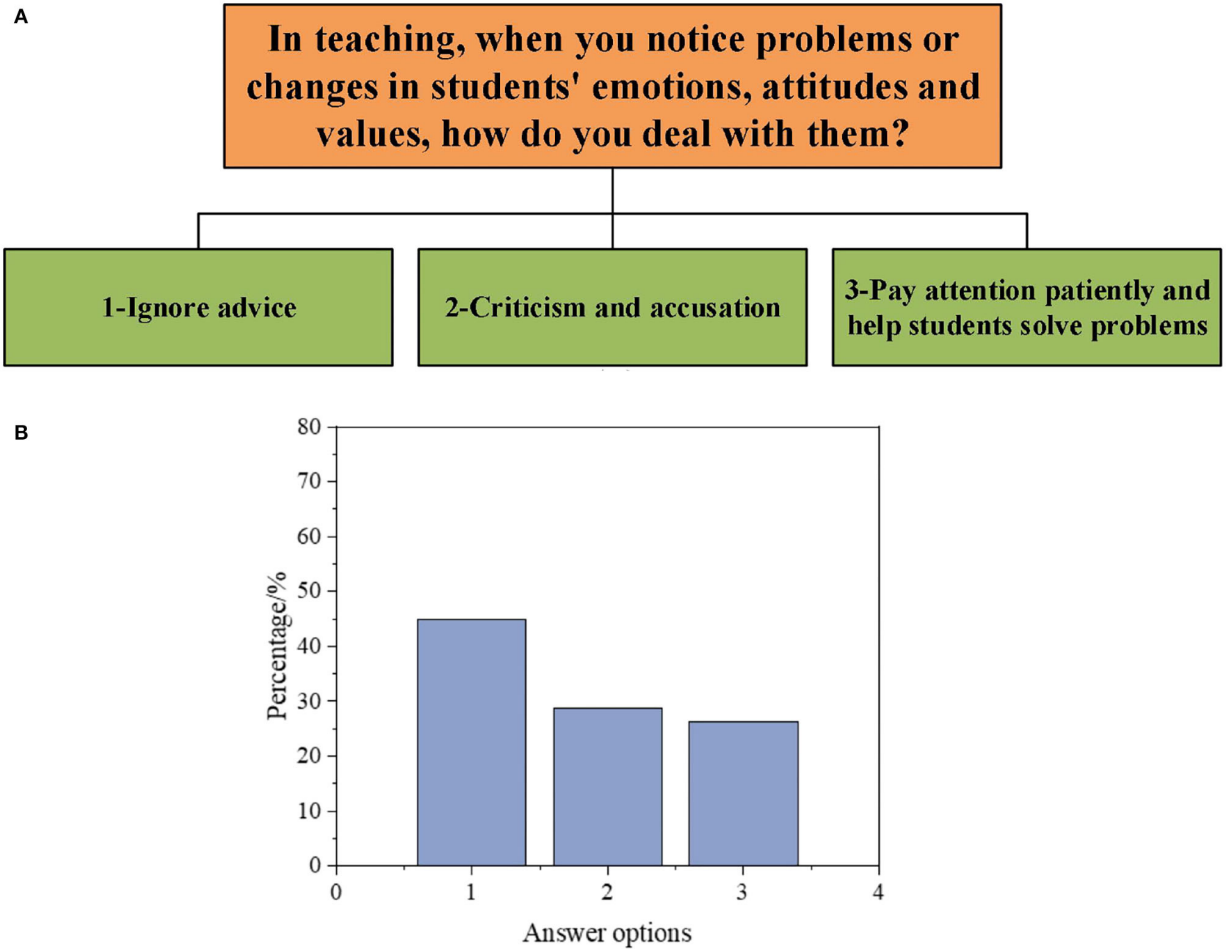
Interview data on the DAU of students' psychological experience-oriented GCR [**(A)**: questions and options; **(B)**: proportion of options].

**Figure 5:**
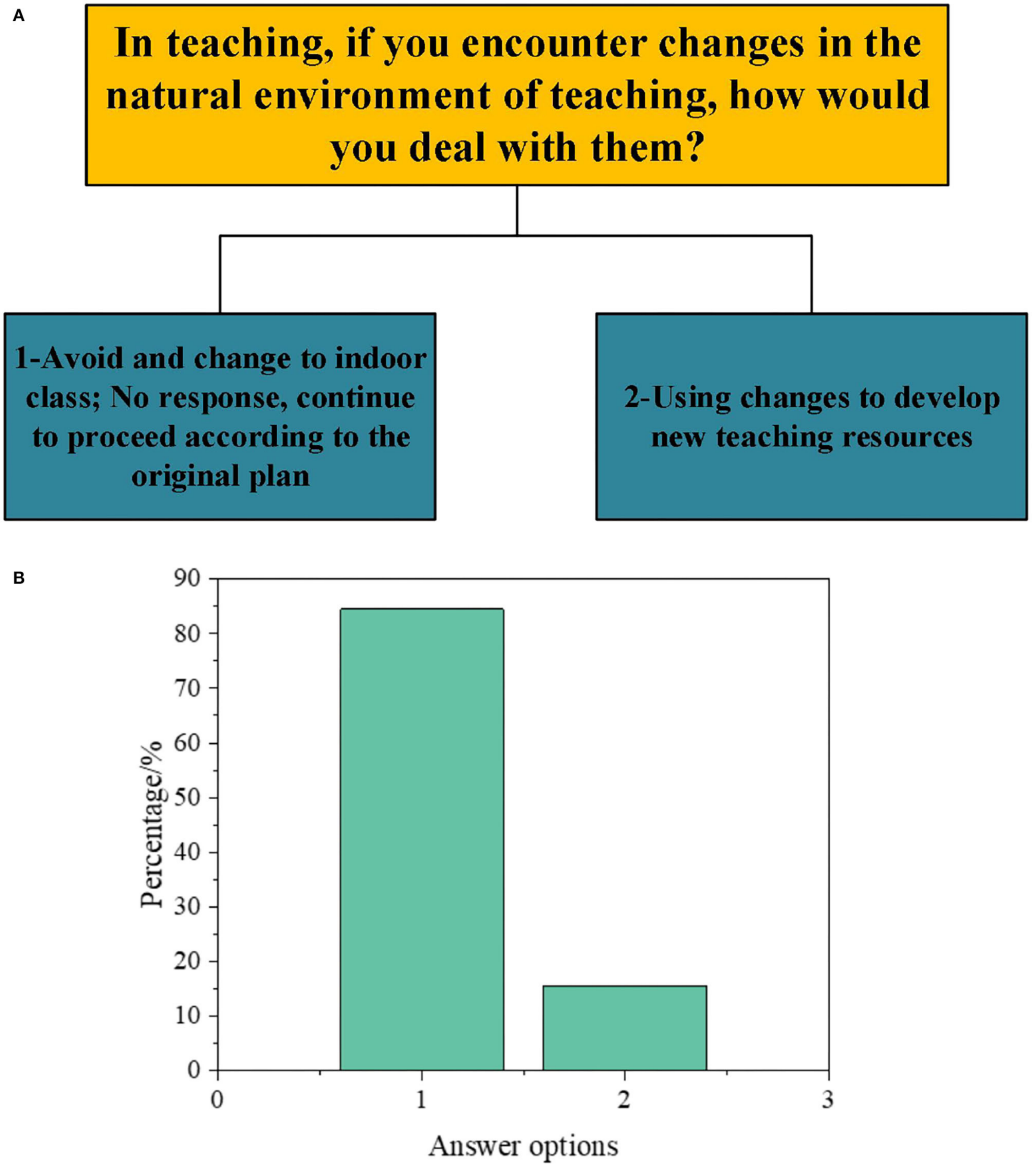
Interview data on the DAU of natural environment-oriented GCR [**(A)**: questions and options; **(B)**: proportion of options].

Subsequently, to understand the DAU of different types of GCR by PE teachers, the results are obtained according to a series of questions in the interview, as illuminated in Figures 1–5.

As depicted in Figure 1, when facing students' error GCR, 71.1% of teachers directly correct errors through explanation or demonstration, and 15.8% of teachers help correct errors through other methods. Teachers seldom ignore students' errors GCR.

As shown in Figure 2, when facing students' individual differences, 47.3% of the teachers help those with low original level or poor learning ability, 31.5% of the teachers choose to ignore the difference, 13.2% of the teachers criticize and accuse students, and only 7.8% of the teachers select customized teaching according to the individual differences.

As outlined in Figure 3, when faced with students' different suggestions, Of this, 28.9% of teachers ignore them. About 39.5% of teachers judge, criticize, or immediately deny. And 28.9% of teachers respect students' views and lead students back to the standard answer. Additionally, a few teachers act according to the specific situation to decide whether to take students' suggestions.

As demonstrated in Figure 4, in the face of students' psychological changes, such as emotions, attitudes, and values, 28.9% of teachers criticize students or immediately deny their views. About 26.3% of teachers can pay attention to students and patiently help students solve problems, but many teachers ignore them.

As displayed in Figure 5, when facing the natural environmental changes in teaching (not strong enough to hinder the teaching), only 15.5% of teachers use the change to develop new teaching resources. Most teachers adhere to the original plan.

Before the “DR” policy, teachers' GCR DAU behaviors differ largely. However, on students' error-oriented GCR, no teachers choose the DAU method of ignoring. Most teachers will focus on criticism or ignore (bypass) when developing and utilizing resources other than students' errors and individual differences.

#### Problems in the DAU of GCR in Primary School PE

**Figure 6:**
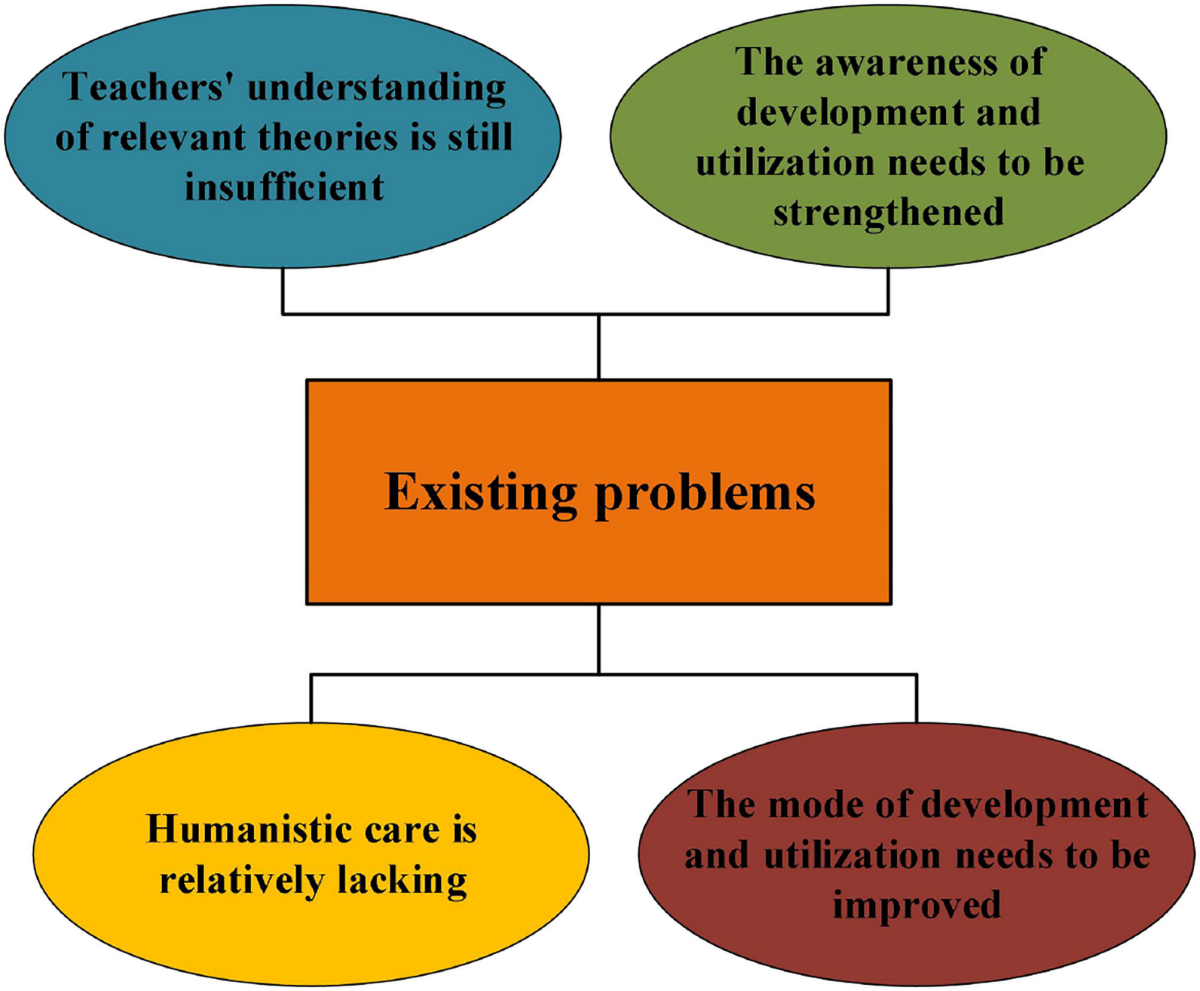
Problems in the GCR DAU of primary school PE.

Figure 6 sketches teachers' interview results of the problems existing in the DAU of GCR in primary school PE. In the current teaching, teachers' DAU of GCR is often accidental. At the same time, the lack of understanding of GCR and related theories will also affect teachers' choice of DAU methods. At present, PE teachers mainly focus on the GCRDAU that can directly benefit students' sports skills. They ignore the GCR that may develop students' emotions, attitudes, and values. Specific DAU strategies can improve teachers' awareness of the GCR DAU and provide guiding ideas for teachers.

### Descriptive Statistics

After the “DR” policy, schools have a rich choice of extra-curriculum classes. As enumerated in Table 3, science, calligraphy and painting, and sports rank among the top three in the number of choices. Since everyone can participate in multiple extra-curriculum classes, the proportional sum is >1. According to the data survey, the students and parents who choose to participate in the off-campus sports class are more than those in the off-campus music class and slightly less than the off-campus science and painting class. The results reflect the students' and parents' preference for the extra-curriculum sports class and their attention to physical exercise.

**Table 3:** Participation in non-discipline extra-curriculum classes.

**Variable**	**Category**	**Percentage/%**
Participation in non-discipline off-campus classes (multiple choices)	Sports	18.9
	Calligraphy and painting	20.7
	Music	15.8
	Science	24.7
	Foreign language	11.3
	Brain development	7.2
	Other	10.4
	Non-participation	31.3

**Figure 7:**
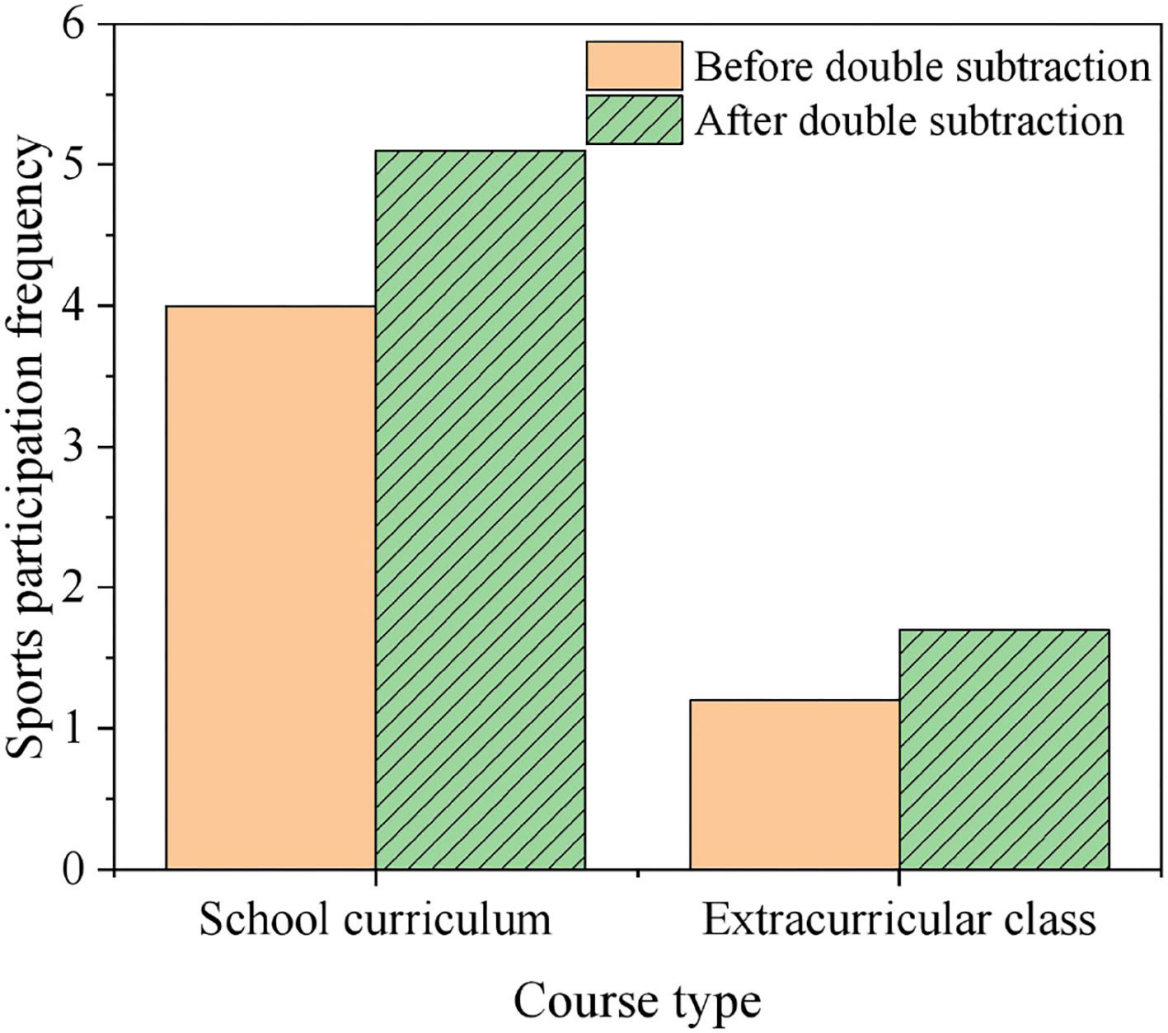
SP frequency before and after “DR”.

Figure 7 compares the frequency of on-campus SP before and after “DR” (since a single class in primary school extends about 40 min, 40-min minimum organized sports activities are recorded as SP).

As revealed in Figure 7, after the “DR”, the on-campus and off-campus SP frequency has increased. According to the interview with teacher W of T school, T school has adjusted the original sports class from 4 to 5 times a week. It aims to improve the students' physical exercise intensity. Additionally, as charted in Figure 7, the SP frequency of sports off-campus classes has also increased by 75%. The time and funds released due to the cancellation of discipline training have also been used for off-campus sports training. In addition, the survey found that before and after the DR, 36 and 28% of parents said that they did not know their children's SP frequency. The results prove that the relationship between parents and the school needs to be strengthened. Thus, it shows that parents lack attention to their children's PE.

**Figure 8:**
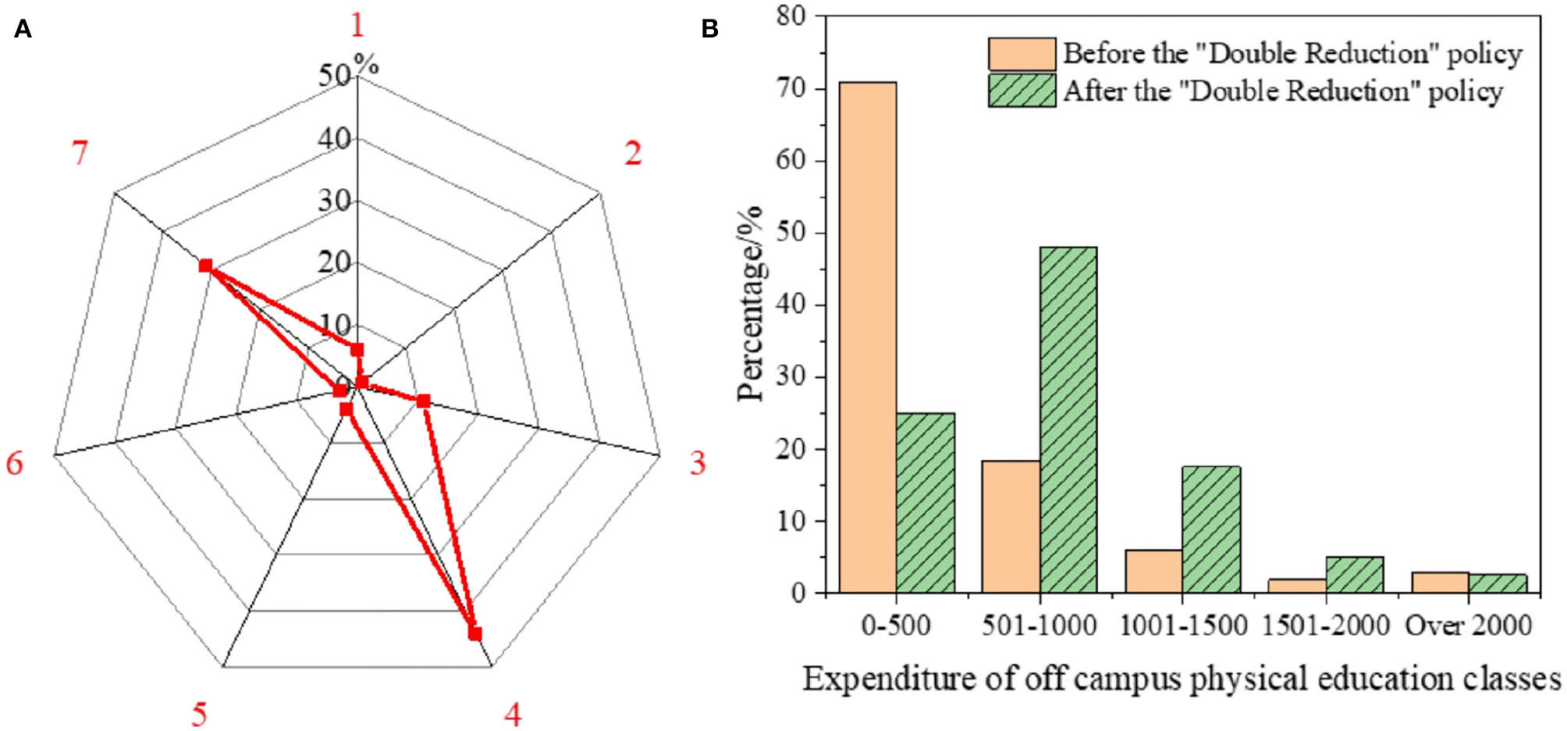
Statistical results of registration purpose and cost of off-campus sport class [**(A)**: proportion distribution of registration purpose of off-campus sport class; **(B)**: cost expenditure of off-campus sport class].

In the survey sample, the purpose of signing up for off-campus sports classes is expressed by numbers 1–7. The specific proportion is distributed in Figure 8A. No.1 represents that students thenselves require to enroll in the classes. No.2 represents that just follow surrounding students' choices. No.3 represents for increasing the interpersonal skill of kids. No.4 aims to improve physical health. No.5 means that preparing for high school or college entrance exam. No.6 means other reasons. No.7 aims to develop hobbies. In today's society, families have a deeper understanding of the overall development of children and the education of sports and art, on which they are more willing to spend on their children. The statistical results of the expenditure on off-campus sports classes are plotted in Figure 8B.

As charted in Figure 8A, the primary purpose of parents signing up for sports classes for their children is to exercise their children's bodies and cultivate their interests and hobbies. Only 4% of parents sign up based on preparing for the sports evaluation of the College Entrance Examination (CEE; Gaokao). Hence, the score increase in sports in the CEE has not been as high as the discipline training before the “DR”, which increases parents' anxiety. For example, the interviewed T school teachers have expressed that more parents have become anxious after implementing the “DR” policy. On the one hand, they are worried that their children's academic achievements will not be as well as others in CEE. On the other hand, given richer after-school time, parents have more reasons to worry about their children's possible addiction to the virtual world. Traditional ideas and the modern lifestyle are the main causes of parents' concerns. As suggested in Figure 8B, about the expenditure of off-campus sports classes, regardless of “DR”, families' expenditure is mainly kept within 1,000 RMB per month. However, after the “DR”, the number of families with an expenditure of 0–500 RMB decreases rapidly. The number of families with an expenditure of 501–1,000 RMB and 1,001–1,500 RMB per month is 2.6 times and 2.9 times the corresponding number of families before the “DR”, respectively.

Although all the respondents live in Shenyang and are urban students, the sixth National Venue Survey data bulletin shows 962,700 sports venues in cities and towns, with a venue area of 1.337 billion square meters. In comparison, there are only 679,700 sports venues in villages, with a venue area of 612 million square meters. Thus, sports facilities in cities far exceed that in rural areas, and urban children enjoy more advantageous sports resources than rural students.

### Influence of Family Background on Students' Participation in Off-Campus Sports

This section excludes the respondents who have no idea of off-campus SP frequency based on descriptive statistics. It implements a measurement model to deeply understand the impact of family background on participation in off-campus sports classes. Without specific classification, it takes students' SP frequency in off-campus sports classes. Based on this, a linear regression model is built for the situation before and after “DR”. The classified variable and parental occupation are set as dummy variables. Table 4 specifies the variables and results.

**Table 4:** Multiple regression analysis (MRA) results of off-campus class SP frequency.

**Category**	**Variable**	**SP frequency of off-campus sports class before “DR”**	**SP frequency of off-campus sports class after “DR”**
		**Beta**	**Beta**
	Gender (reference level is female)	−0.16***	−0.14***
	Number of children in the family (the reference level is the only child)	−0.13*	−0.14*
	Father's years of education	0.048	0.092
	Mother's years of education	0.043	0.036
	Annual household income	0.22***	0.33***
Father's occupation	Workers or the unemployed	0	0
	Service personnel or individual industrial and commercial households	1.03	2.01
	Civil servants of party and government organs, personnel of enterprises and institutions, or technicians	1.53*	1.83*
	Teachers, engineers, doctors, or lawyers	1.25*	2.15*
Mother's occupation	Workers or the unemployed	0	0
	Service personnel or individual industrial and commercial households	0.22	0.93
	Civil servants of party and government organs, personnel of enterprises and institutions, or technicians	0.60	0.73
	Teachers, engineers, doctors, or lawyers	0.24	0.96
	*N*	956	
	*R* ^2^	0.12	0.15

*Indicates significant at 0.05 level. **Means significant at 0.01 level. ***Represents significant at 0.001 level.

In terms of individual factors, students' gender significantly affects the off-campus SP frequency. Regardless of “DR”, boys' off-campus SP frequency is higher than girls'. This is partly caused by boys' physical structure and interests. On the other hand, parents hope that sports can make boys more masculine. Additionally, the number of family children has a significant negative impact on students' off-campus SP frequency. The more children there are in a family, the less likely they will participate in off-campus sports.

Family income has a significant impact on the off-campus SP frequency. Regardless of “DR”, the higher the family income is, the more students participate in off-campus sports. Off-campus sports classes can cultivate students' comprehensive quality. As shown in the statistics on parents' motivation to sign up for Off-campus sports classes for their children, parents are mainly intended to cultivate interests and hobbies and exercise their children's bodies. This off-campus counseling is considered an “icing on the cake” behavior. Therefore, family income has a great impact on the frequency of participation.

The parents' years of education have no significant effect on students' off-campus SP frequency. The empirical research reveals that the higher the family income and parents' education level are, the higher the children's off-campus SP frequency is. Possibly, previous studies have involved all sports activities, while the off-campus sports classes reported here only consider some charging items. Therefore, family income greatly impacts students' off-campus SP frequency, and the impact of parents' years of education can be ignored. Put differently, the impact of family income is too prominent, and the impact of parents' years of education is weak and no longer significant.

The father's occupation significantly affects students' off-campus SP frequency, while the mother's occupation has no significant effect. Children of CPC and government officials, employees of enterprises and institutions, and technicians have a significantly SP frequency than children of workers. Specifically, the gap is 1.53 and 1.83 units before and after the “DR”, respectively. Meanwhile, children of teachers, engineers, doctors, and lawyers present a 1.25 and 2.15 higher off-campus SP frequency than workers' children before and after the “DR”, respectively. Probably, the income of intellectual occupations is relatively stable and rich in social resources. Signing up for off-campus sports classes is consumption behavior, so income is strong support. In addition, intellectuals have a high social status and pay more attention to their children's quality education.

### Sports Category Selection Behavior

Further, to explore the opening and selection of on-campus and off-campus sports before and after the “DR”, this section investigates students' SP by considering family incomes. Because the QS is designed with a multi-choice form for sports classes, the category selection of off-campus classes is repeated. Table 5 lists the distribution of the proportion of students choosing different off-campus sports types. Because many people choose more than one sport, the sum is >1.

**Table 5:** Distribution of off-campus sports class selection after “DR” under different family income.

**Category**		**Football**	**Basketball**	**Tennis**	**Badminton**	**Indoor** **skating/** **skiing**	**Fencing**	**Table tennis**	**Taekwondo**	**Dance**	**Other**	**Non**
Family income	Maximum 25%	3.8%	25.6%	0.8%	13.5%	7.5%	0.8%	6.0%	4.5%	23.3%	5.6%	24.1%
	Upper middle 25%	5.0%	28.8%	1.4%	7.2%	8.6%	0.0%	9.4%	7.9%	17.3%	6.1%	20.9%
	Lower middle 25%	6.5%	30.6%	0.8%	5.6%	2.4%	0.0%	4.8%	11.3%	16.9%	4.7%	29.0%
	Minimum 25%	9.1%	21.7%	0.0%	4.3%	0.0%	0.0%	9.1%	3.5%	22.2%	5.5%	37.0%
	Chi square	4.7	6.1	2.6	8.35*	21.4*	4.7	1.2	3.7	2.7	-	91.1**

*Indicates significant at 0.05 level. **Means significant at 0.01 level. ***Represents significant at 0.001 level.

Table 5 uncovers the selection of sports types in off-campus sports classes. Basketball, dance, taekwondo, and football are very popular in families of all income levels. There is a large market demand in these areas. The overall participation rate of indoor skating/skiing, fencing, and tennis is meager, and they are concentrated in the class with high family income. Such projects belong to minority sports in China, with high-class fees, which is more suitable for families with high family income and attention to children's international education. The chi-square test between the four types of family income and each sport shows that badminton and indoor skating/skiing have significant differences among families with different incomes. There are significant differences among families without off-campus classes. Children from families with the top 50% of household income have more opportunities to participate in off-campus sports classes.

### Choice Behavior of On-Campus Sports Activities

Table 6 unveils that on-campus sports activities mainly involve common projects, without great innovation, such as basic track and field, football, basketball, badminton, and table tennis. However, after the “DR”, various sports activities in the on-campus sports class involve a more comprehensive range of students. The school is encouraging students to experience more kinds of sports activities. Additionally, the number of students involved in other sports has increased. According to the QS results, after the “DR” policy, new sports are added to the “rope skipping” and “aerobics”, such as “Spread Fight” and “Tai Chi Fan”. The school actively promotes “DR” with sports empowerment. However, according to the interview with school teachers, the current sports projects are still relatively limited, and there are too few PE teachers. The school is currently discussing cooperation with off-campus PE training institutions to introduce more excellent and professional teachers and better hardware. The aim is to maximize the effectiveness of “school-enterprise cooperation”. There is no regression analysis for the SP frequency of on-campus sports activities at the individual and family levels. It is because on-campus sports activities mainly involve PE and physical activity classes, which are compulsory and have little correlation with individual and family factors.

**Table 6:** SPon-campus sports activities before and after “DR”.

**On-campus sports**		**Basic track and field**	**Football**	**Tennis**	**Badminton**	**Basketball**	**Table tennis**	**Taekwondo**	**Dance**	**Other**
Before DR	Participant	532	94	6	25	268	135	18	73	29
	Proportion	55.6%	9.8%	0.6%	2.6%	28.0%	14.1%	1.9%	7.6%	3.0%
After DR	Participant	554	113	14	29	341	139	25	106	69
	Proportion	57.9%	11.8%	1.5%	3.0%	35.7%	14.5%	2.6%	11.1%	7.2%

## Discussion

The “DR” is a protracted war. The existing difficulties need the joint efforts of families, schools, and the government. Parents are the first-hand teachers of their children. Parents need to change traditional ideas and avoid exam-oriented thinking. Besides, they should establish correct ideas, guide their children to exercise, invest in their children to participate in sports activities, and support and understand school sports. Moreover, parents should take the initiative to reduce their excessive concerns about academic performance. Physical exercise and subject performance are not contradictory but complement each other. A good physique guarantees long-term development, the evaluation of which has been included in the CEE. Still, parents should avoid short-term examination thinking (Otero-Saborido et al., 2021; Santos and Monteiro, 2021).

Therefore, schools should pay practical attention to cultivating students' physical fitness and sports quality and expand PE teaching projects. This can help avoid the expansion of inequality in SP caused by family status. Further, for projects with expensive venues or coaches, the government can give appropriate subsidies to encourage high-quality off-campus training to enter the campus. Lastly, an excellent physical education class in school can avoid the unfair phenomenon of educational resources brought by family class, which focuses on subject training (Meng et al., 2021).

The government should continue to reduce the burden through continuous policy guidance. Introducing sports scores in the education assessment can avoid the solid examination-oriented nature by strengthening the value-added evaluation and process evaluation of PE, rather than just the result evaluation. Additionally, there is a need to accelerate the homogeneity of PE in regional and school differences and promote educational equity. At the same time, the gap between urban and rural areas should be bridged. The differences in the frequency and quality of off-campus sports class participation caused by economic conditions should be further narrowed.

## Conclusion

This study collects students' on-campus and off-campus SP in Shenyang through a QS and interviews under the background of “DR”. Then, it explicitly analyzes the development and utilization of the Generative Resources in primary school sports. At the same time, it also focuses on the impact of family background on children's participation in off-campus sports classes under the control of personal factors and school factors. Based on this, the reality of the current difficulties is understood. After the “DR”, parents and schools gradually pay attention to student's physical health and better understand their on-campus physical exercise. However, research findings also reveal some problems, such as in sufficient communication between parents and school. Regardless of “DR”, some parents do not know the type and frequency of sports their children carry out in school. Some parents have anxiety, which is rooted in the traditional PE concept under the long-term exam-oriented education in China.

After “DR”, the consumption expenditure of off-campus sports classes has increased significantly. The motivation of parents to sign up for sports off-campus classes for their children is mainly to exercise and cultivate interest. A few parents also sign up because of the increasing weight of PE in the college entrance examination. Students' on-campus and off-campus SP types and frequency have increased. Schools and parents have paid more attention to students' physical quality. Popular sports, such as football, basketball, table tennis, badminton, and taekwondo, maintain a high degree of participation both in on-campus and off-campus classes. After the “DR”, although the number of participants in fencing, indoor skiing/skating, and other small and high-cost projects has increased slightly, the participation is still low. The diversity of sports projects in the school is insufficient. Presumably, the policy landing time is still relatively short. There is not enough time to adjust the venue and teacher capital. The PE collaborative teaching with off-campus institutions has not been on the right track. Lastly, there is a slight shortage of teachers during the period of overweight PE. The boys' off-campus SP is significantly higher than the girls' at the individual level due to physical characteristics and natural hobbies.

In terms of family background, family income and the father's occupation significantly affect the children's off-campus SP frequency. The impact of parents' years of education is not significant. Due to the charging nature of off-campus classes, wealthy families are more likely to obtain counseling resources. PE is a part of quality education. As the driving force for the upward mobility of the lower class, education alleviates or changes the inequality caused by birth and shapes inequality. The survey shows that wealthy and stable-income families will take advantage of economic resources to ensure that their children have more physical training opportunities. The distribution of opportunities among families of different social classes is unequal, making sports off-campus training bend toward the rich. There is no doubt that the comprehensive quality of sports plays a vital role in individual development. The strong position of advantageous social strata in comprehensive quality training may further enhance their comprehensive competitive advantage. The shortcomings of this work are: first, the number of samples is relatively small. Secondly, the comparison is not very obvious because the implementation time of “DR” policy is relatively short. After the long-term implementation of the “DR” policy, the follow-up work needs to explore the frequency, project types, and off-campus activity expenditure of primary school students in combination with GCR utilization in primary schools in other cities. It is hoped to improve the SP of primary school students further.

## Data Availability Statement

The raw data supporting the conclusions of this article will be made available by the authors, without undue reservation.

## Ethics Statement

Ethical review and approval was not required for the study on human participants in accordance with the local legislation and institutional requirements. Written informed consent from the patients/participants legal guardian/next of kin was not required to participate in this study in accordance with the national legislation and the institutional requirements.

## Author Contributions

SL: design the questionnaire, conduct the survey, and write and revise the paper. GW: revise the questionnaire, conduct the survey, and design and revise the paper. Both authors contributed to the article and approved the submitted version.

## Funding

This study received funding from the Second Batch of Industry-University Cooperation Collaborative Education Project, Ministry of Education of PRC: Construction of Children's Exercise Guidance Micro-video and Sharing Course (Grant No. 202102277001), and Liaoning Province Education Science and Research Department Young Scientific and Technological Talents Nurturing Project: Research on Multiple Supply Models of Youth Public Sports Services from the Perspective of Actor Network (Grant No. SK2020116).

## Conflict of Interest

The authors declare that the research was conducted in the absence of any commercial or financial relationships that could be construed as a potential conflict of interest.

## Publisher's Note

All claims expressed in this article are solely those of the authors and do not necessarily represent those of their affiliated organizations, or those of the publisher, the editors and the reviewers. Any product that may be evaluated in this article, or claim that may be made by its manufacturer, is not guaranteed or endorsed by the publisher.
